# Implementation of a Data Packet Generator Using Pattern Matching for Wearable ECG Monitoring Systems

**DOI:** 10.3390/s140712623

**Published:** 2014-07-15

**Authors:** Yun Hong Noh, Do Un Jeong

**Affiliations:** Division of Computer & Information Engineering, Dongseo University, 47 Jure-ro, Sasang-gu, Busan 617-716, Korea; E-Mail: noh108@dongseo.ac.kr

**Keywords:** wearable, ECG, pattern matching, data packet generator, abnormal heartbeat, smartphone

## Abstract

In this paper, a packet generator using a pattern matching algorithm for real-time abnormal heartbeat detection is proposed. The packet generator creates a very small data packet which conveys sufficient crucial information for health condition analysis. The data packet envelopes real time ECG signals and transmits them to a smartphone via Bluetooth. An Android application was developed specifically to decode the packet and extract ECG information for health condition analysis. Several graphical presentations are displayed and shown on the smartphone. We evaluate the performance of abnormal heartbeat detection accuracy using the MIT/BIH Arrhythmia Database and real time experiments. The experimental result confirm our finding that abnormal heart beat detection is practically possible. We also performed data compression ratio and signal restoration performance evaluations to establish the usefulness of the proposed packet generator and the results were excellent.

## 1. Introduction

Heart disease is still a global threat worldwide. Preventive healthcare comes into the discussion where if someone could continuously monitor his or her heart activity state and by alerted by instant alarm feedback, heart disease could potentially be screened for at the very early stage. Therefore, wearable, clothing type, unconstrained systems for measuring routine ECG signals has been studied tremendously in the literature [1–4]. However, a continuous and wireless ECG monitoring solution requires active transmission for data analysis. Large amounts of data transmission over a long time could eventually cause transmission bottlenecks, increased delays and cost. Therefore, techniques for reducing the amount of transmit data for continuous ECG measurement is required. There are literatures which have presented several data techniques for raw ECG data compression to reduce the amount of data and a compression ratio of 5 to 20:1 is achieved [5]. The compressed data is transmitted and can convey heath information for real-time monitoring on a PC or smartphone or store it on a server through characteristic ECG point detection (QRS segment, P wave, ST segment and T wave) and abnormal heartbeat classification. Thus, if only the minimum necessary information after performing the characteristic ECG point detection and abnormal heartbeat classification within the ECG measuring system is transferred, rather than transmitting the whole raw ECG data, the amount of the data packets to transmit can be greatly reduced

A lot of existing research related to characteristic point detection and abnormal heartbeat classification has been studied. In previous studies, ECG characteristic point detection involved detecting each of the feature points (*i.e.*, QRS segment, P wave, ST segment and T wave) by applying some algorithm such as rule-based, wavelet, genetic, DTW, HMM, *etc.*, and calculating the spacing between each feature point and width [6–11]. However if there is morphological distortion such as abnormal heart signal or a noise contaminated signal, the correct position of the characteristic point will be out of place and thus making the characteristic point detection accuracy very low. In addition, there are studies for abnormal heartbeat or rhythm classification using fuzzy, neural network, SVM, K-mean clustering and phase space algorithms [12–19], but when comes into real time processing, the previously mentioned algorithms need to extract multiple parameters in parallel with the signal processing and perform real time recursive calculations. Therefore, the demands on the system memory for real time processing are relatively high and practically impossible for a mobile system. Therefore, research to reduce amount of real time data transmitted through a simple abnormal heart detection method is highly recommended.

In this paper, we counter the abovementioned problem by deploying a packet generator in our in-house designed wearable ECG monitoring system. The packet generator is implemented by using a pattern matching technique to minimize the amount of information to be transmitted, serving the purpose of effective and fast data transmission. Data packets are sent to mobile device via Bluetooth and are extracted and reconstructed to a normal ECG pattern using our own Android application. In short, the advantage of the proposed packet generator is to create enveloped information in a very small size data packet that is sufficient to reconstruct and analyze ECG signals at the end user side (mobile phone).

## 2. Implementation of Data Packet Generator Using Pattern Matching

### 2.1. Wearable ECG Monitoring System

**Figure 1.**
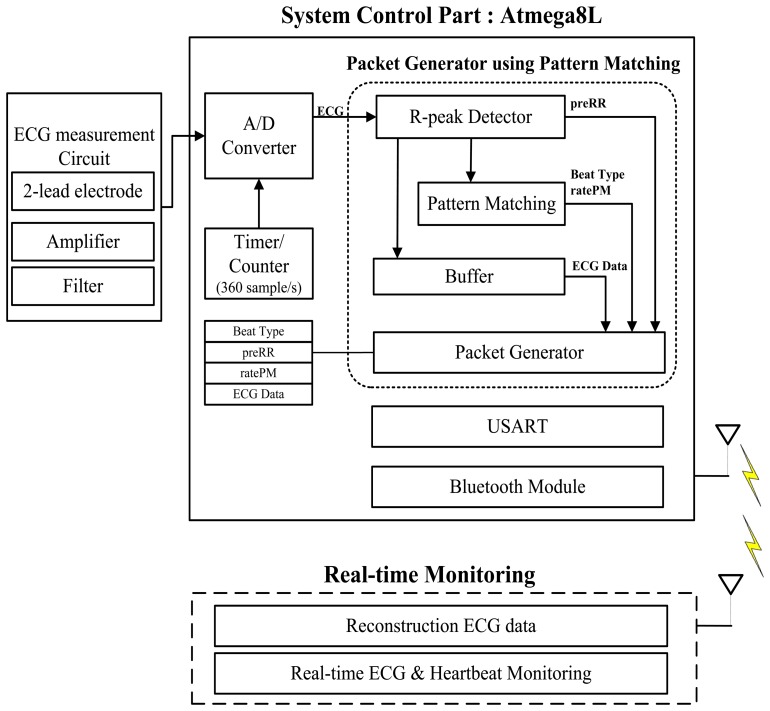
Overall view of the wearable ECG monitoring system with an embedded packet generator.

A data packet generator is proposed to minimize the amount of data packet transferred. In previous studies [20], we presented a wearable ECG monitoring system and in this paper, we further enhance the system by installing our proposed data packet generator within it. Figure 1 shows the overall view of the wearable ECG monitoring system.

The ECG measurement circuit consists of 2-lead ECG electrode, amplifiers and filter circuit. The ECG system is using an ATmega8L microcontroller which is well-suited for low power operation. The system converts analog ECG signal to digital data. A Bluetooth v2.0 module ESD200 (Parani™, SENA, Inc., Seoul, Korea) is used as a wireless transceiver. A pattern matching technique is implemented for heart rate detection and abnormal heart beat detection. Pattern matching parameters such as preRR, ratePM, and buffered ECG data are then enveloped into a single packet using the data packet generator. The packet generator minimizes the weight of data packet created and sends only minimum information for health information analysis. The data packet that is transferred to a smartphone through Bluetooth transmission and signal can be restored and monitored in real-time using our Android application.

### 2.2. Pattern Matching Algorithm

**Figure 2.**
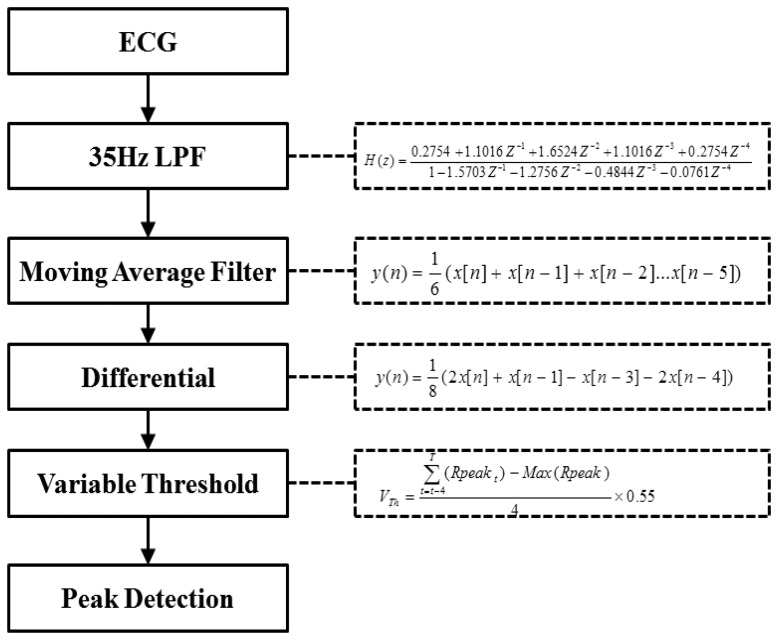
Block Diagram of the R-peak detection process.

In this paper, a pattern matching algorithm is embedded into the system control for real time R-peak detection and abnormal heartbeat detection. The R-peak detection process consists of several simple formulas as shown in Figure 2. A 35 Hz low pass filter is used to remove high frequency noise. The moving average filter is used for removal of power noise. First derivative of ECG signals is implemented to enhance the R-peak component and finally a variable threshold formula is applied to detect the R peak.

**Figure 3.**
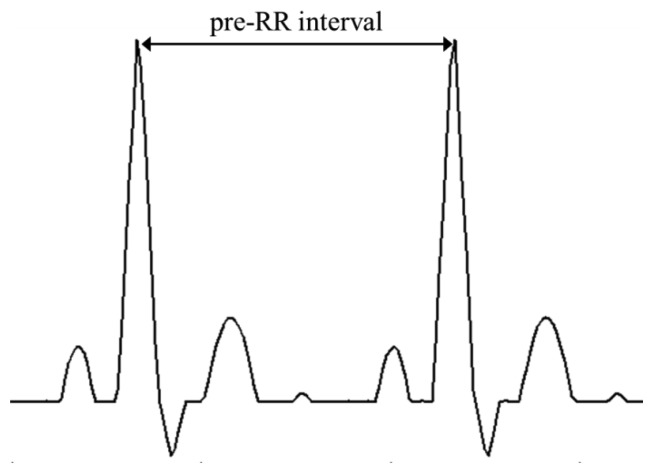
Example of calculation preRR.

The detected R-peak is used to calculate the preRR and ratePM which are parameter for detection of abnormal heartbeat. Figure 3 shows preRR is the time interval between the R-peak. ratePM is a parameter that reflects morphological abnormalities.

Arrhythmia classification for tachycardia and bradycardia can be easily determined by measuring the R-peak–R-peak (preRR) interval. However, measurement of R-peak–R-peak interval does not reflect the morphological features of the ECG (QRS segment, P wave, ST segment and T wave) from the abnormal cardiac activity. Therefore, we implement a simple real-time abnormal heartbeat algorithm using pattern matching to reflect the morphological features.

The pattern matching algorithm is a matrix pattern matching method. First, matrix pattern for normal heartbeat is generated. Next the real time input ECG signal is compared with the matrix pattern. The operation of matrix pattern comparison can be easily implemented in a limited system.

In this pattern matching algorithm, R-peak is the first and initial parameter to be detected. From the point of the R peak, we identify the PQRST area by setting a window on an appropriate time interval. The PQRST window start at 220 ms before the R-peak occurrence and we name the starting point of the window as PQRST-on and the window will end at 380 ms after the R-peak occurrence and we name the ending point of the window PQRST-off. In other words, a full PQRST area will gives a total sample of 80 (PRQ samples) + 1 (R-peak sample) + 137 (RST samples) = 218 samples.

The first 30 seconds of normal heartbeat data is collected. Then, each of the PQRST areas is determined and extracted for pattern generation. Instead of using 10-bit ADC data for PQRST pattern extraction, we use only 5-bit, thus reducing the computation complexity during micro-processing. The extracted PQRST pattern is 218 × 81 in resolution. This PQRST pattern is use to generate a normal ECG matrix pattern for the use of abnormal heartbeat detection.

The extracted PQRST patterns are overlapped with each other. The overlapping in matrix pattern is counted on a matrix cell basis. Initially, each of the matrix cell values start at 0. With every one additional overlapping on the same matrix cell, the cell value is increased by 1. The maximum cell value of a cell matrix is set to be 10. If the overlapping cell value reached its maximum value of 10, there will be no further increment of the cell value, and hence this particular matrix cell is finalized as a perfectly matched cell. With the above mentioned recursion steps, we finally generate a matrix pattern for normal heartbeat.

**Figure 4.**
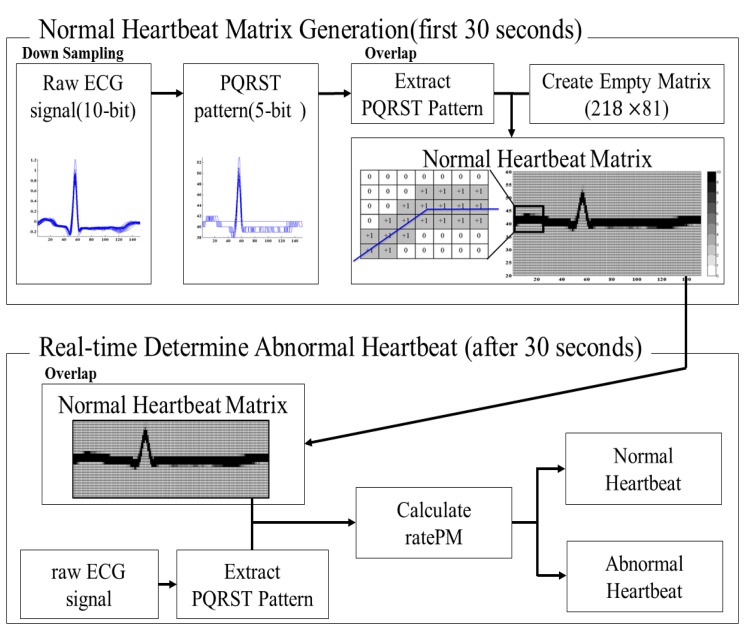
Process of creating a normal heartbeat matrix.

ECG matrix pattern generation is used to classify the abnormal heartbeat from the normal heartbeat by matching with matrix pattern. After 30 s of ECG recording a normal heartbeat matrix is generated. Then, the microprocessor will start to prepare the ECG matrix pattern to compare with the real time input pattern while detecting the R-peak (the PQRST pattern is extracted while detecting the R-peak and the PQRST pattern is meant to be the matrix pattern). The overlapping matrix pattern value is set arbitrarily. If the overlapping input ECG matrix cell value reaches a value of more than 7, then the particular cell it is counted as ratePM = 1. In other words, if there is a perfectly match of input ECG matrix cell value, the final ratePM will be equal to 218. The abnormal heartbeat classification threshold is set at ratePM = 180. If final ratePM is greater than 180, this means the heart beat is normal, whereas if the final ratePM is less than 180, then the heart beat is classified as abnormal. The threshold setting is to increase the tolerance band to adapt to any minor variation of amplitude and phase in a normal heartbeat. Therefore, the occurrence of false alarms is significantly reduced. In short, if the overlapping matrix pattern is matched with the pre-created pattern, then the ratePM appears to be high. If the matrix overlapping is out of the match with matrix pattern, then the ratePM appears to be low. The process of creating a matrix for normal ECG signals is as shown in Figure 4. The simplified Matlab pseudo code for matrix generation is as shown below:

IF PM(i, PW(i, j)) < 10 → PM(i, PW(i, j)) = PM(i, PW(i, j)) + 1
IF PM(i, PW(i, j) −1) < 10 → PM(i, PW(i, j) –1) = PM(i, PW(i, j) –1) + 1
IF PM(i, PW(i, j) + 1) < 10 → PM(i, PW(i, j) + 1) = PM(i, PW(i, j) + 1) + 1
PW = PQRST Pattern
PM = Normal Heartbeat Matrix

**Figure 5.**
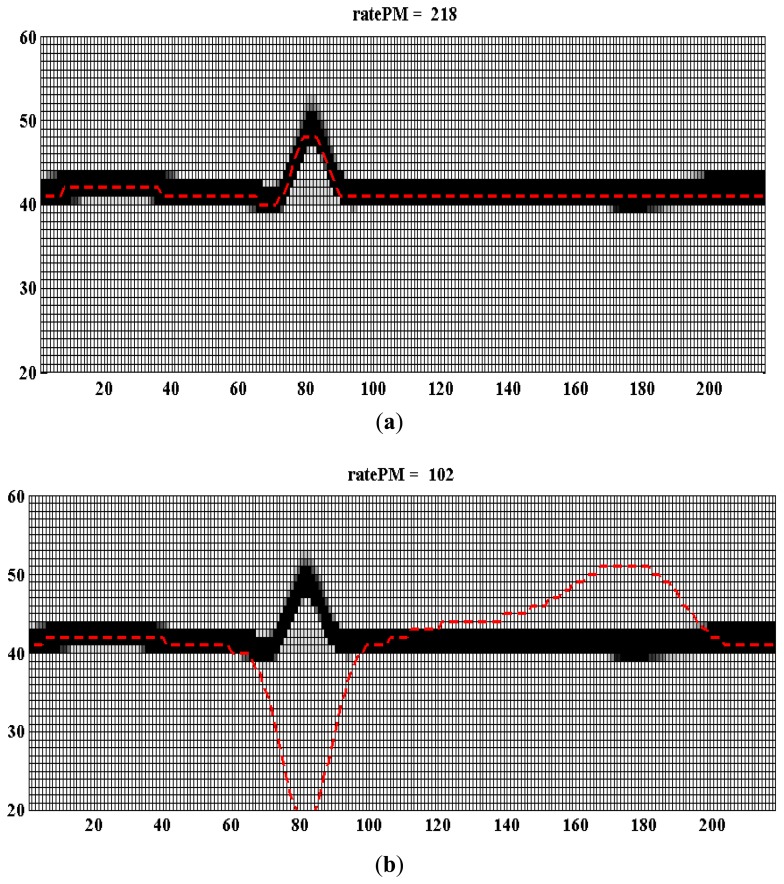
Example of normal heartbeat and abnormal heartbeat to determine. (**a**) Normal heartbeat; (**b**) Abnormal heartbeat.

Figure 5 shows an example of applying the proposed abnormal heartbeat detection algorithm. By observing Figure 5a the normal heartbeat shows a high ratePM of 218. But in Figure 5b an abnormal ECG only shows a low ratePM of 102. The proposed algorithm is embedded inside the control system of the wearable ECG measurement system.

### 2.3. Data Packet Generator

A packet generator generates a packet variably according to a normal heartbeat and an abnormal heartbeat and transmits beat type, RR interval (preRR), and ratePM ECG data. If it is classified as an abnormal heartbeat, it will transfer the entire packet including ECG data including the very first packet of the ECG pattern.

**Figure 6.**
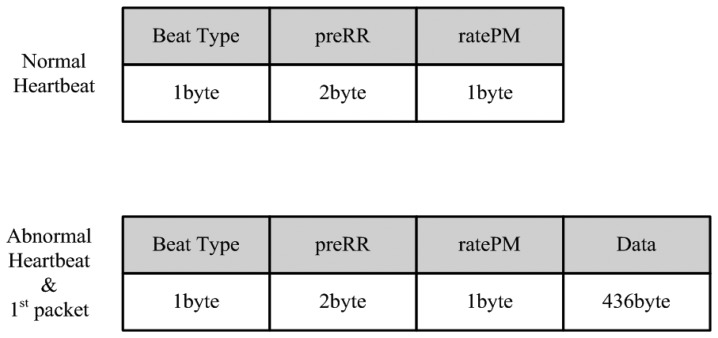
Data packet structure of packet generator.

In the case of normal heartbeat, a data packet with only beat type, preRR and ratePM is created and transmitted instead of ECG data. This is because the normal heartbeat is classified as duplicate data and has similar form to the very first generated matrix pattern. By using the trick of not always transmitting the full amount of data, the possibility of data packet collision at the receiver side is minimized. Figure 6 shows the data packet structure of the packet generator.

**Figure 7.**
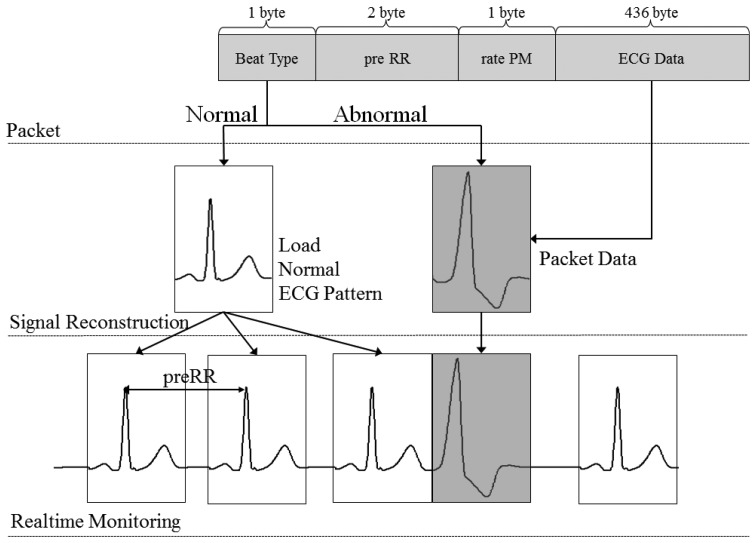
Process of ECG signal reconstruction.

The real-time ECG monitoring application identifies the wireless transmitted data packet by checking the beat type. If the beat type is N, then the application classified this is a normal heart beat and reloads the pre-created normal ECG matrix to the screen. If the beat type is A, then the receiving beat is classified as an abnormal heart beat. The entire ECG data within the data packet will be loaded to the screen. Figure 7 show the process of ECG signal reconstruction.

## 3. Experimental and Results

### 3.1. Evaluation of Pattern Matching Algorithm

**Figure 8.**
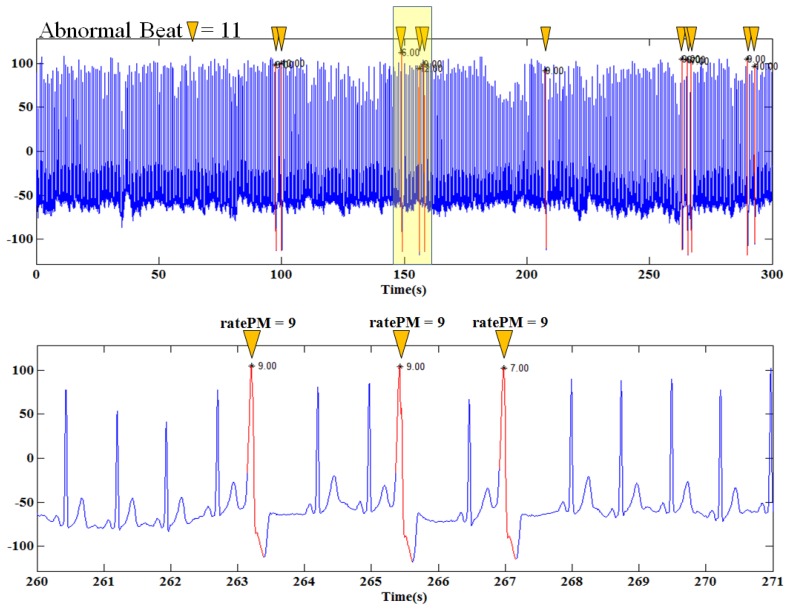
Example of abnormal heartbeat detection using the MIT/BIH Arrhythmia Database 116 record.

In this paper, we evaluate the performance of the pattern matching algorithm using the MIT/BIH Arrhythmia Database. Figure 8 shows an example of detected abnormal ECG using the MIT/BIH Arrhythmia Database 116 Record. This record contains 345 heartbeats and 11 arrhythmias. If ratePM of 35 and below is detected, the ECG is classified as an abnormal ECG. The abnormal ECG is marked by an * mark. This 116 Record shows 100% detection for arrhythmia. Through the example of Figure 8, the pattern matching algorithm using calculated ratePM for abnormal heartbeat detection is visually confirmed to be practical.

A total of seven MIT/BIH Arrhythmia Databases sampled at 360 Hz are used to evaluate the performance of the proposed pattern matching algorithm. The evaluation result is shown in Table 1. The result comparison in Table 1 shows that the average accuracy of R-peak detection is 99.9% while the abnormal ECG detection accuracy is 98.3%, which are relatively high and almost perfect. The MIT/BIH arrhythmia database used in the experiment is patients' data which contains serious arrhythmias (including the annotation of arrhythmia and abnormal heartbeat). In the case of an R-peak detection error due to serious arrhythmias and not showing a big difference with a normal pattern (although corresponding to an abnormal heart rate as classified by the annotation), the arrhythmia heartbeat fails to be detected. The setup of ratePM will have an impact on detection performance due to trade-off and thus 100% detection accuracy could not be achieved.

### 3.2. Experimental of Data Packet Transmission

**Figure 9.**
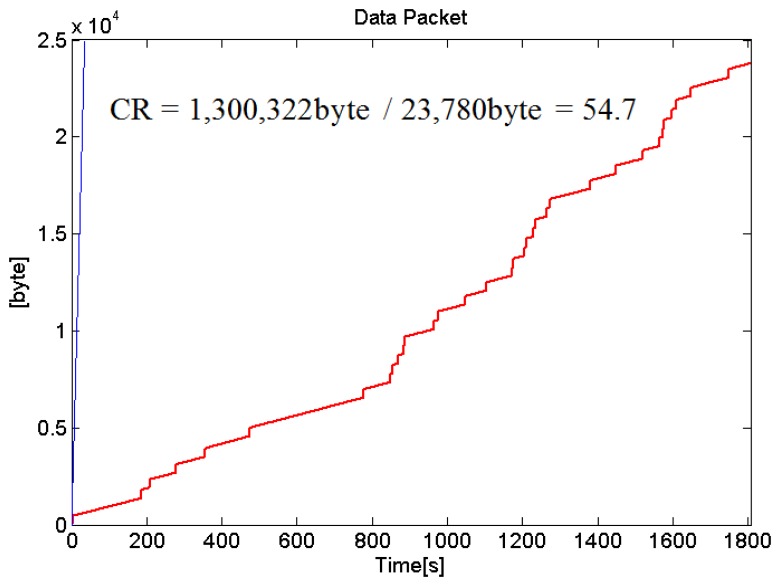
Comparison plot of data packet created by comparing convention data packet generator and our proposed data packet generator.

Real-time ECG monitoring system is implemented and evaluated. The full amount of data will be transmitted if an abnormal heartbeat is detected whereas if a normal heart beat is detected, only minimal information such as heart rate and activity status will be transmitted. In order to simulate the situation where there is a detection of abnormal heart rate, MIT/BIH Arrhythmia Database 100 record is loaded into the ECG measurement system for experimental purposes. The ECG measurement system transmits the data packets to the smartphone in real time. Conventionally, if we send and receive a single data packet, it will take up 2 bytes per 10 bit sample. Without implementing our proposed packet generator, with a 30 min of MIT/BIH Arrhythmia Database 100 record, a conventional packet generator will send a total of 650,161 samples data and take up to 1,300,322 bytes for the entire wireless transmission. After implementing our proposed packet generator, only 23,780 bytes are generated and sent. This is because we only send the first packet of the ECG pattern, and send abnormal ECG data only when there an alert is triggered. Figure 9 shows the comparison plot of data packet generated by comparing our data packet generator with a convention data packet generator. It shows very good performance in terms of compression ratio (CR) of 54.7:1. The data compression ratio is calculated using Equation (1):

(1) $$\text{Compression Ratio}\;(\text{CR})=\frac{\text{total raw data packets}}{\text{total transmitted data packets}}$$

The existing ECG lossless or lossy compression algorithm has a CR of 5–20:1 [5]. The existing papers transmit compressed raw ECG data and tend to restore and analyze it at the receiver side. Therefore, their approach is different from our proposed data packet generator.

**Figure 10.**
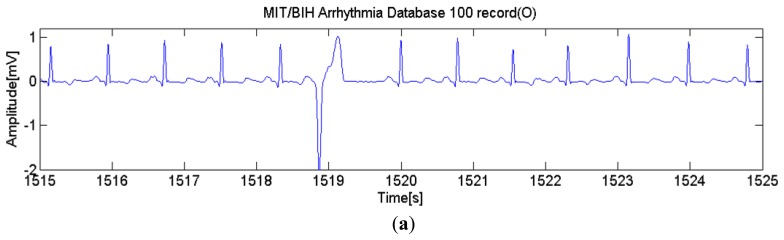

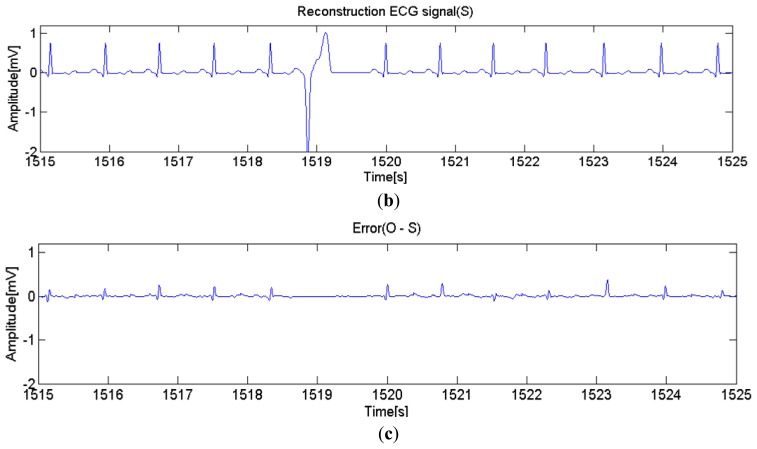
Example of restoration signal of a MIT/BIH 100 record: (**a**) is the raw signal; (**b**) is the reconstructed signal; and (**c**) is the error of (a) and (b).

Data packets are transmitted and received at the mobile device in real time. Initially, a normal ECG matrix pattern is generated and sent to the mobile device as its first data packet. This normal ECG matrix pattern will be stored at the mobile device. Whenever a beat type of N data packet is received, the monitoring application will load the ECG matrix pattern and display it as a normal ECG signal on the screen. When another normal beat type data packet is received, the display signal will be reconstructed to connect the abnormal ECG data at the tail, thus showing a continuously varying ECG signal on the display. For an abnormal heartbeat it reconstructs the signal and displays it based on the data that is transmitted. The signal restoration error is evaluated and the result is shown in Figure 10.

Correlation coefficient (CC) and root mean square error (RMSE) between the signals in Figure 10a and b are calculated. The correlation coefficient is as high as 0.964 and RMSE is as low as 0.039. Therefore, the proposed data packet generator is verified to be an effective method for wireless transmission.

In order to further evaluate the overall performance, another eight records are selected from MIT/BIH Arrhythmia Database for further assessment. The results of R-peak detection error rate, number of detected abnormal heart beat, compression ratio, correlation coefficient, and root mean square error are shown in Table 2.

### 3.3. Real time ECG monitoring Experiment

**Figure 11.**
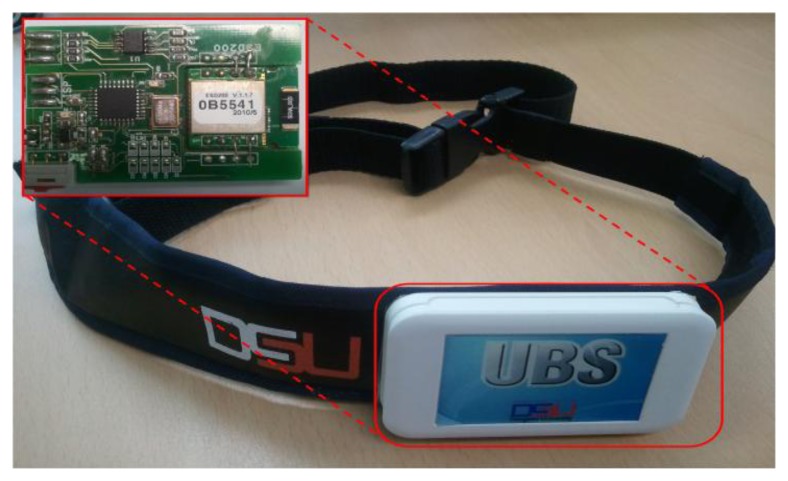
Wearable ECG measurement system.

The wearable ECG monitoring system with built-in pattern matching algorithm was implemented for real time ECG measurement and abnormal heart beat detection. Figure 11 shows the wearable ECG measurement system.

**Figure 12.**
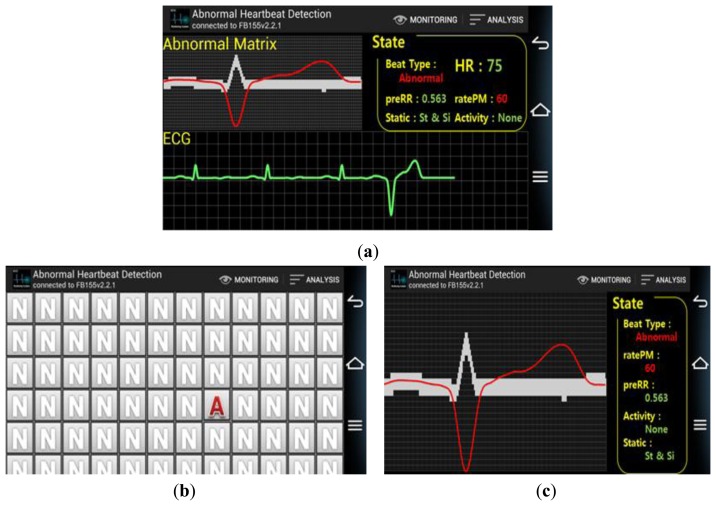
(**a**) Monitoring View State; (**b**) Analysis View State-1; (**c**) Analysis View State-2.

A smartphone-based monitoring application is developed to monitor various parameters like beat type, preRR, ratePM and a provide a graphical presentation of the reconstructed ECG signal. Several analysis view states are provided by the application as shown Figure 12. Monitoring view states (Figure 12a) is the default page to present real time compact information like abnormal matrix, real time ECG, and received data packet information such as beat type, HR, preRR, ratePM, *etc.* Analysis View State-1 (Figure 12b) and -2 (Figure 12c) are the summary of the health analysis. Analysis View State-1 shows the entire beat type in a matrix map presentation, whereas Analysis View State-2 shows the specifically abnormal ECG pattern overlapping with the normal ECG pattern.

A real time ECG monitoring experiment is set up to evaluate the performance of the proposed solution. The objective of the experiment is to demonstrate a practical scenario of daily life ECG monitoring. A Physio Lab P400v commercial ECG measurement device [21] is used as a control source for the real time experiment. The R-peak detected and the total amount of data recorded by the P400 are tabulated. The P400 is not a mobile ECG device. Therefore, tests for a moving scenario are excluded in this experiment.

**Figure 13.**
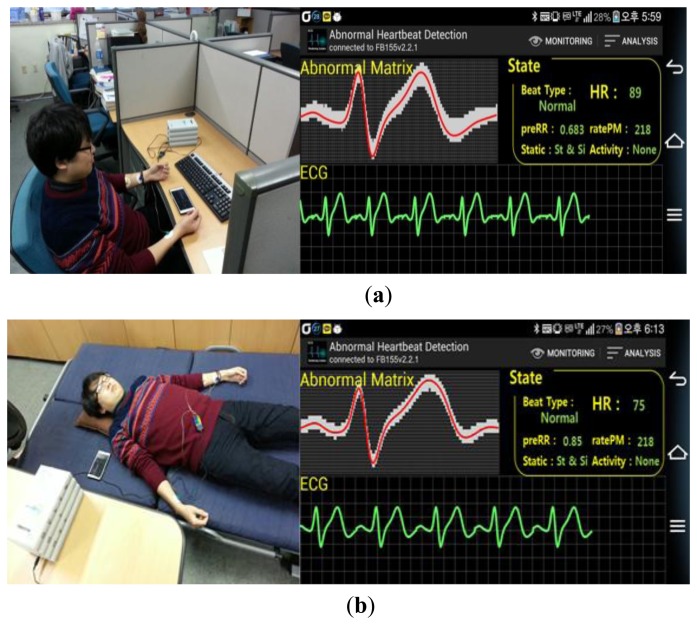
Experiment scenario. (**a**) Sitting scenario; (**b**) Sleeping scenario.

The user is requested to wear the wearable ECG monitoring system and the external connection for the Physio Lab P400 is set up. A total of 30 min of simultaneous ECG measurement using thye proposed system and the P400 are performed. Two test scenarios are simulated: sitting scenario and sleeping scenario. The test scenario is repeated on five different subjects. Figure 13a demonstrates the sitting scenario where a user is wearing the wearable ECG system together with an external attachment with the P400 whereas Figure 13b demonstrates a sleeping scenario where a user is wearing the wearable ECG monitoring system together with the external attachment with the P400. Throughout the experiment, the author is monitoring his ECG signal using a Galaxy3 smartphone equipped with Android version 4.0.4 (Ice Cream Sandwich).

Assuming the R-peak detection of the commercial Physio Lab P400 ECG measurement is genuine, we compare our wearable ECG monitoring system total detected R-peak with the commercial P400 one. On the other hand, we also compare the amount of data recorded in the P400 with our system. The data compression ratio is calculated using Equation (1). The experimental result is shown in Table 3.

## 4. Conclusions

A wearable ECG monitoring system was implemented to monitor ECG signals in daily life. In addition, a packet generator that applied a pattern matching algorithm was implemented for the detection of abnormal heartbeats in real-time. A packet generator can minimize the amount of data packets to transmit by sending only the minimum information needed for health analysis. ECG data is restored at the smartphone and heart activity status is monitored.

A performance evaluation of the data transfer rate and data restoration rate is performed and evaluated. The restored signal shows an average similarity of 0.95 compared to the original signal and giving a very low average error of only 0.08. An extreme result of 176.48 compression ratio is achieved in record 123. This is because there are only three abnormal heart beats in the record. The contrary explanation applies to record 106 which gives a relative low compression ratio of 5.59 due to the large number of abnormal heart beat counts. A real time ECG measurement experiment is set up to confirm our findings. As a result, we achieve a promising average R-peak detection accuracy of 99.8%. Furthermore, the total data recorded on a P400 unit is significantly larger than the total data recorded in the wearable ECG monitoring system and thus we achieve a very high average compression ratio of 193.9. The experimental results show that the amount of data packets is reduced significantly, but in order to check the validity of abnormal heartbeat detection, clinical tests on actual patients may be necessary.

Therefore, the objective of practical implementation of wearable ECG monitoring system to screen and monitor patients' ECG signals has been achieved in our paper. In other words, the data packet generator reduces packet generation through pre-analysis. In case of low occurrence of abnormal heartbeats, it offers a great advantage of data packet reduction. In future research, algorithm optimization to periodically/adaptively update the normal ECG template depending on the time/state (activity) and optimal ratePM threshold calculation are the next challenge. Some pre-processing techniques such as normalization techniques, and strong HPF (1 Hz) might be introduced to reduce motion artifacts caused by movement. Finally, a comparative study with existing ECG compression algorithms in the same environment and clinical tests on real patients as experimental subjects could be the next move.

## Figures and Tables

**Table 1.** Results of R-peak detection and abnormal heartbeat detection.

Record	MIT/BIH		Pattern Matching Algorithm		R-Peak Detection Accuracy (%)	Abnormal Detection Accuracy (%)
	
	Total Beat	Arrhythmia	R-Peak	Abnormal		

100	2273	1	2272	1	99.9	100
119	1987	444	1987	439	100	98.9
121	1863	1	1861	1	99.9	100
123	1518	3	1518	3	100	100
124	1619	47	1619	52	100	89.4
230	2256	1	2256	1	100	100
231	1573	2	1571	2	99.9	100

					99.9	98.3

**Table 2.** Results of experimental data packet transmission.

Record	Total Beat	R-Peak Detection Error Rate (%)	Number of Abnormal Heartbeat	Abnormal Detection Accuracy (%)	CR	Signal Restoration	

						CC	RMSE

102	2187	0.09	4	100.00	124.12	0.85	0.09
103	2084	0.00	2	100.00	141.33	0.97	0.06
106	2027	0.05	520	99.42	5.59	0.95	0.09
112	2539	0.04	2	100.00	118.00	0.95	0.04
116	2412	0.46	109	99.08	22.75	0.93	0.20
123	1518	0.07	3	99.93	176.48	0.97	0.06
215	3363	0.00	165	89.09	15.12	0.97	0.06
221	2427	0.12	396	98.99	7.19	0.98	0.06

Avg.						0.95	0.08

**Table 3.** Results of real time ECG monitoring experiment.

Subject		Time [s]	R-Peak	R-Peak Detection Accuracy (%)	Number of Abnormal Heartbeat	CR	Signal Restoration	

							CC	RMSE

1	Sitting	1,863	2,822	100	1	164.98	0.94	0.04
	Sleeping	10,409	12,879	100	4	206.97	0.95	0.06

2	Sitting	1,826	2,496	100	2	176.21	0.97	0.05
	Sleeping	11,645	12,203	100	12	232.21	0.92	0.03

3	Sitting	1,706	2,074	100	2	194.70	0.93	0.08
	Sleeping	11,714	15,420	99.5	8	191.64	0.98	0.07

4	Sitting	1,716	2,310	100	0	189.50	0.92	0.05
	Sleeping	11,242	13,614	99	9	205.96	0.94	0.04

5	Sitting	1,782	2,415	99.9	2	177.24	0.95	0.05
	Sleeping	11,777	14,872	99.9	7	200.46	0.99	0.06

Average				99.8		193.99	0.95	0.05

## References

[1] Bourdon L., Coli S., Loriga G., Taccini N., Gros B., Gemignani A., Cianflone D., Chapotot F., Dittmar A., Paradiso R. (2005). First Results with the Wealthy Garment Electrocardiogram Monitoring System. Comput. Cardiol..

[2] Anliker U., Ward J.A., Lukowicz P., Troster G., Dolveck F., Baer M., Keita F., Schenker E.B., Catarsi F., Coluccini L. (2004). AMON: A wearable multi parameter medical monitoring and alert system. IEEE Trans. Inform. Technol. Biomed..

[3] Bifulco P., Gargiulo G., Romano M., Fratini A., Cesarelli M. (2007). Bluetooth Portable Device for Continuous ECG and Patient Motion Monitoring during Daily Life. IFMBE Proc. Medicon..

[4] Ishijima M. (1993). Monitoring of electrocardiograms in bed without utilizing body surface electrodes. IEEE Trans. Biomed. Eng..

[5] Mukhopadhyay S.K., Mitra S., Mitra M. (2012). An ECG signal compression technique using ASCII character encoding. Measurement.

[6] Adam G., Witold P. (2003). A Genetic Segmentation of ECG Signals. IEEE Trans. Biomed. Eng..

[7] Joao P.V., Madeiro P.C., Cortez F.I., Oliveira R.S. (2007). A new Approach to QRS Segmentation based on Wavelet bases and Adaptive Threshold Technique. Med. Eng. Phys..

[8] Natalia M.A., Deng Z.D., Poon C.-S. (2008). Anlysis of First-Derivative Based QRS Detection Algorithms. IEEE Trans. Biomed. Eng..

[9] Ivaylo I.C. (2004). Real time elecrocardiogram QRS detection using combined adaptive threshold. BioMed. Eng. Online.

[10] Ali Z., Sohrab S., Mohammad H., Moradi F.T. (2006). Automated ECG Segmentation Using Piecewise Derivative Dynamic Time Warping. Int. J. Biolog. Med. Sci..

[11] Rodrigo V., Andreao B., Dorizzi J.B. (2006). ECG Signal Analysis through Hidden Markov Models. IEEE Trans. Biomed. Eng..

[12] Hu Y., Palreddy S., Tompkins W.J. (1997). A patient-adaptable ECG beat classifier using a mixture of experts approach. IEEE Trans. Biomed..

[13] de Chazal P., Duyer M.O., Reilly R.B. (2004). Automatic classification of heartbeat using ECG morphology and heart beat interval features. IEEE Trans. Biomed. Eng..

[14] T.Ince S.K., Gabbouj M. (2009). A generaric and robust system for automated patient-specific classification of ECG signals. IEEE Trans. Biomed. Eng..

[15] Ge D., Srinivasan N., Shankar M.K. (2002). Cardiac Arrhythmia Classification using Autoregressive Modeling. Biomed. Eng. OnLine.

[16] Lim J.S. (2009). Finding Features for Real-Time Premature Ventricular contraction detection Using a Fuzzy Neural Network System. IEEE Trans. Biomed. Eng..

[17] Douglas A.C., Richard M.S. (1990). An Approach to Cardiac Arrhythmia Analysis Using Hidden Markov Models. IEEE Trans. Biomed. Eng..

[18] Awadhesh P., Manabendra B. Wavelet and Energy Based Approach for PVC Detection.

[19] Jalal A.N., Mostafa S.H., Sadoghi Y., Mahmoud N., Bahram N. Intelligent Arrhythmia Detection using Genetic Algorithm and Emphatic SVM(ESVM).

[20] Yap J.H., Noh Y.H., Jeong D.U. (2012). The Deployment of Novel Techniques for Mobile ECG Monitoring. Int. J. Smart Home.

[21] PhysioLab http://www.physiolab.co.kr.

